# Scientometric Study of Mpox and Vaccine Research: Dynamics, Emerging Patterns, and Networking

**DOI:** 10.1002/hsr2.70443

**Published:** 2025-03-09

**Authors:** Fran Espinoza‐Carhuancho, Juan Alvitez, Abigail Temoche, Victor Roman‐Lazarte, Frank Mayta‐Tovalino

**Affiliations:** ^1^ Academic Department, Human Medicine Career Universidad Nacional Federico Villarreal Lima Peru; ^2^ Academic Department, Research, Innovation and Entrepreneurship Unit Universidad Nacional Federico Villarreal Lima Peru; ^3^ Escuela de Posgrado Universidad Continental Lima Peru; ^4^ Vicerrectorado de Investigación Universidad San Ignacio de Loyola Lima Peru

**Keywords:** bibliometrics, global public health, Mpox, vaccine

## Abstract

**Background and Aim:**

Mpox (Monkeypox) is a zoonotic disease transmitted through contact with infected animals or humans. Recent research focuses on epidemiology, transmission, and vaccine development to combat its resurgence. This bibliometric study analyzed the dynamics, emerging patterns, and networks of mpox and vaccine research from 2019 to 2024.

**Methods:**

The literature search was conducted in the Scopus database on August 18, 2024, initially identifying 1278 papers published between January 2019 and August 2024. A specific search strategy was applied to collect documents. The retrieved documents were analyzed using the Scival and Bibliometrix tools to obtain bibliometric metrics. Data analysis was performed using R Studio and Scival.

**Results:**

During the study period, 562 sources were identified that contributed to 1278 papers on mpox and vaccine research. These papers show an impressive annual growth rate of 91.49%. Several institutions stood out for their contributions to mpox and vaccine research. The journal Vaccines had 58 publications, followed by the Journal of Medical Virology and Vaccine with 31 publications each. According to Bradford's law, in Zone 1, the journal Vaccines had 58 publications. According to Lotka's law, most authors in the field of mpox and vaccine research have written only one article.

**Conclusion:**

These findings highlight the diversity of sources contributing to the mpox and vaccine research literature and highlight the importance of these sources in terms of their scholarly impact and relevance to the research community. This study provides valuable insight into emerging trends and patterns in the field.

## 1 Introduction

Mpox was previously an endemic disease in Central and West Africa, but in recent years, it has expanded globally [1]. Between 2017 and 2018, a significant outbreak occurred in Nigeria [2]; cases were also reported in the United Kingdom, Israel, and Singapore [3]. From January 2022 to April 2024, the World Health Organization (WHO) recorded 95,226 cases in 117 countries, leading to the declaration of this outbreak as a global health emergency due to the notable increase in cases [4].

Mpox, also known as monkeypox, is a zoonotic disease caused by the mpox virus, which belongs to the orthopoxvirus family. It is transmitted mainly through direct contact with infected animals, such as rodents and primates, or through close contact with infected people. Symptoms include fever, headache, muscle aches, lymphadenopathy and a characteristic skin rash that progresses to pustules and crusts [1, 2, 3, 4, 5]. The disease is generally self‐limiting, and its symptoms last from 2 to 4 weeks. It is characterized by fever and skin lesions, with lymphadenopathies being the most striking; however, there are severe cases that can lead to death. Severity varies according to virus strain, with mortality rates between 1% and 10% [5]. Individuals with HIV or no previous smallpox vaccination are especially vulnerable. Men who have sex with men (MSM) or bisexual men, as well as those with sexually transmitted infections, are at high risk of infection [6]. Of the cases registered between 2022 and 2024, 85.5% occurred in MSM, and 51.9% were also coinfected with HIV. This implies that approximately 50% of patients with mpox are at risk of serious complications [7]. Given the global scope of the outbreak and the severity of some cases, it is important to better define how to control this epidemic.

In the absence of specific treatments, vaccine prevention is essential for the public health response to mpox. Because of its antigenic similarity to the vaccinia virus, the smallpox vaccine offers cross‐protection [8]. In the recent outbreak, men aged 18–44 years were the most affected, accounting for 79.4% of cases [9]. Therefore, there is a need to learn more about vaccines against this pathology since the most affected population tends to be young people and adults. The efficacy of the smallpox vaccine to prevent mpox has been estimated to be between 35% and 85%, with few adverse effects, and some countries have approved its emergency use in this context [10]. In this sense, it is necessary to know those populations, countries, and approaches to vaccine research on this disease.

A bibliometric analysis allows us to map scientific production on a topic of interest. Knowing the emerging topics, countries, and authors that have contributed more about Mpox vaccines would help us to understand the information gaps, as well as to direct the information according to research trends. Therefore, this study analyzed the dynamics, emerging patterns, and networks of mpox and vaccine research from 2019 to 2024.

## 2 Methods

### 2.1 Ethical Approval

This scientometric study did not involve humans or animals; therefore, ethical approval was not required.

### 2.2 Criteria Selection

All types of documents published between January 2019 and August 2024 that addressed topics related to mpox and vaccines were included, using a specific search formula in the Scopus database. These documents covered articles, reviews, letters, letters, notes, book chapters, editorials, errata, short surveys, conference papers, retractions, and books. On the other hand, documents that did not meet the established temporal or thematic criteria were excluded, as well as those that were not available in English. In addition, duplicate studies and those that did not provide complete or relevant data for bibliometric analysis were discarded.

### 2.3 Study Design and Literature Search

The study was descriptive using a bibliometric approach. The literature search was performed using the Scopus database on August 18, 2024. We initially identified 1278 papers published between January 2019 and August 2024. The papers included 825 articles, 286 reviews, 68 letters, 42 notes, 16 book chapters, 12 editorials, 11 errata, 8 short surveys, 8 conference papers, 1 retraction, and 1 book.

### 2.4 Search Formula

The following search strategy was applied in Scopus: TITLE‐ABS (“monkeypox” OR “Monkeypox virus” OR “Human monkeypox” OR “Orthopoxvirus infection” OR “MPX” OR “MPXV” OR “mpox”) AND TITLE‐ABS (“vaccine” OR “Immunization” OR “Vaccination” OR “Serum” OR “Prophylactic vaccine”) AND PUBYEAR > 2018 AND PUBYEAR < 2025.

### 2.5 Analysis in SciVal and Bibliometrix

In SciVal, document analysis functionality was used to examine the bibliometric metrics of the retrieved documents. This included the number of publications, citations, authors, keywords, and cross‐country collaborations. Graphs and tables were generated to visualize these metrics, and trends over time were identified. In Bibliometrix, a deeper analysis of the data were performed. The retrieved documents were imported in bible format, and a document‐terms matrix was created. This matrix was used to perform keyword co‐occurrence analysis, which identified keywords that appeared together frequently. Network analysis was also performed to examine collaborations between the authors and the countries. Network maps were generated to visualize the collaborations.

### 2.6 Data Analysis

Data analysis was performed using R Studio and SciVal. Several key metrics were examined for each country, including scholarly output, view count, citations per publication, views per publication, CiteScore 2023, and SNIP 2023. These metrics provided detailed insights into the impact and relevance of mpox and vaccine research in each country.

## 3 Results

During the 2019–2024, 562 sources were identified that contributed to 1278 documents on mpox and vaccine research. These documents show an impressive annual growth rate of 91.49%. The average age of the papers was 1.13 years, and each paper received an average of 11.73 citations. In total, 44,286 of these papers were referenced. The papers contained 2048 keywords provided by the authors. A total of 7982 authors contributed to these papers, with 72 authors producing single‐author papers. On average, each paper had 8.47 coauthors, and 31.46% of the papers included international coauthorships (Table 1).

**Table 1:** Main characteristics.

Description	Results
Timespan	2019:2024
Sources	562
Documents	1278
Annual Growth %	91.49
Document Average Age	1.13
Average citations per doc	11.73
References	44,286
Author's Keywords (DE)	2048
Authors	7982
Authors of single‐authored docs	72
Single‐authored docs	80
Co‐Authors per Doc	8.47
International co‐authorships %	31.46
Article	825
Book	1
Book chapter	16
Conference paper	8
Editorial	12
Erratum	11
Letter	68
Note	42
Retracted	1
Review	286
Short survey	8

During the study period, several institutions stood out for their contribution to monkeypox and vaccine research. The Centers for Disease Control and Prevention in the United States led with 53 publications, which garnered 554 views and an average of 25.7 citations per publication. Harvard University and Johns Hopkins University, also in the United States, contributed 29 and 27 publications, respectively. Emory University in the United States and King Saud University in Saudi Arabia each produced 22 and 21 publications, respectively. In the United Kingdom, University College London and the UK Health Security Agency each contributed 21 and 18 publications, respectively. The University of New South Wales in Australia and Tribhuvan University in Nepal were also significant contributors with 21 and 20 publications, respectively (Table 2).

**Table 2:** Top 10 most representative institutions.

Institution	Country	Scholarly output	Views count	Citations per publication	Views per publication
Centers for Disease Control and Prevention	United States	53	554	25.7	10.5
Harvard University	United States	29	396	11.1	13.7
Johns Hopkins University	United States	27	613	21.5	22.7
Emory University	United States	22	346	30.4	15.7
King Saud University	Saudi Arabia	21	349	12.8	16.6
University College London	United Kingdom	21	509	56.4	24.2
University of New South Wales	Australia	21	219	16.3	10.4
Tribhuvan University	Nepal	20	733	14.3	36.6
Chinese Academy of Sciences	China	18	242	5.9	13.4
UK Health Security Agency	United Kingdom	18	357	48.2	19.8

The journal Vaccines led with 58 publications, a CiteScore 2023 of 8.9, 12.3 citations per publication, and a SNIP 2023 of 0.93. The Journal of Medical Virology and Vaccine followed with 31 publications each, with CiteScores 2023 of 23.2 and 8.7, respectively. The journal Viruses contributed 27 publications, while Morbidity and Mortality Weekly Report contributed 24 publications and had the highest CiteScore 2023 of 65.4 and the highest SNIP 2023 of 6.51. Other major sources included The Lancet Infectious Diseases, Emerging Infectious Diseases, Eurosurveillance, Journal of Infection and Public Health, and Frontiers in Public Health. These results highlight the diversity of sources contributing to the literature on monkeypox and vaccine research, as well as the importance of these sources in terms of scholarly impact and relevance to the research community (Table 3).

**Table 3:** Top 10 most productive scientific journals.

Scopus Source	Scholarly output	CiteScore 2023	Citations per publication	SNIP 2023
Vaccines	58	8.9	12.3	0.93
Journal of Medical Virology	31	23.2	13.8	1.19
Vaccine	31	8.7	15.1	1.08
Viruses	27	7.3	14.6	0.88
Morbidity and Mortality Weekly Report	24	65.4	37.1	6.51
The Lancet Infectious Diseases	18	60.9	25.2	6.1
Emerging Infectious Diseases	17	17.3	43.8	1.54
Eurosurveillance	17	32.7	11.4	2.34
Journal of Infection and Public Health	16	13.1	13.4	1.23
Frontiers in Public Health	15	4.8	7.7	0.94

Ranjit Sah of Tribhuvan University in Nepal led with 18 publications, an h‐index of 32, 15.8 citations per publication and 39.6 views per publication. Kuldeep Dhama of the Indian Veterinary Research Institute in India and Andrea M. McCollum of the Centers for Disease Control and Prevention in the United States followed with 15 publications each. Alfonso J. Rodriguez‐Morales of the Fundación Universitaria Autónoma de las Américas in Colombia contributed 14 publications. Several authors from IRCCS Istituto per le Malattie Infettive Lazzaro Spallanzani—Rome in Italy, including Fabrizio Fabio Maggi, Andrea Antinori, Valentina Mazzotta and Enrico Girardi, contributed 13, 12, 12 and 11 publications, respectively. Panayampalli Subbian Satheshkumar from the Centers for Disease Control and Prevention in the United States and Anil T. Mangla from DC were also contributors with 12 and 11 publications, respectively (Table 4).

**Table 4:** Top 10 most productive authors.

Author	Affiliation	Country	Scholarly output	h‐index	Citations per publication	Views per publication
Sah, Ranjit	Tribhuvan University	Nepal	18	32	15.8	39.6
Dhama, Kuldeep	Indian Veterinary Research Institute	India	15	81	10.5	15.2
McCollum, Andrea M.	Centers for Disease Control and Prevention	United States	15	40	54.5	19.4
Rodriguez‐Morales, Alfonso J.	Fundación Universitaria Autónoma de las Américas	Colombia	14	63	18.5	47.4
Maggi, Fabrizio Fabio	IRCCS Istituto per le Malattie Infettive Lazzaro Spallanzani ‐ Roma	Italy	13	43	8.1	14.1
Antinori, Andrea	IRCCS Istituto per le Malattie Infettive Lazzaro Spallanzani ‐ Roma	Italy	12	87	7.4	14.1
Mazzotta, Valentina	IRCCS Istituto per le Malattie Infettive Lazzaro Spallanzani ‐ Roma	Italy	12	17	7.4	14.1
Satheshkumar, Panayampalli Subbian	Centers for Disease Control and Prevention	United States	12	21	29.6	18.5
Girardi, Enrico	IRCCS Istituto per le Malattie Infettive Lazzaro Spallanzani ‐ Roma	Italy	11	30	7.8	15.1
Mangla, Anil T.	DC	—	11	10	24.9	6.6

According to Bradford's law, in Zone 1, the journal Vaccines had 58 publications, followed by the Journal of Medical Virology and Vaccine with 31 publications each. Zone 1 included 20 sources with 370 publications. Zone 2 began with the New England Journal of Medicine and ended with Vaccine: X, including 76 sources contributing 359 publications. Finally, Zone 3 began with Pathogens and Global Health and ended with Virologica Sinica, including 80 sources contributing 132 publications.

**Figure 1:**
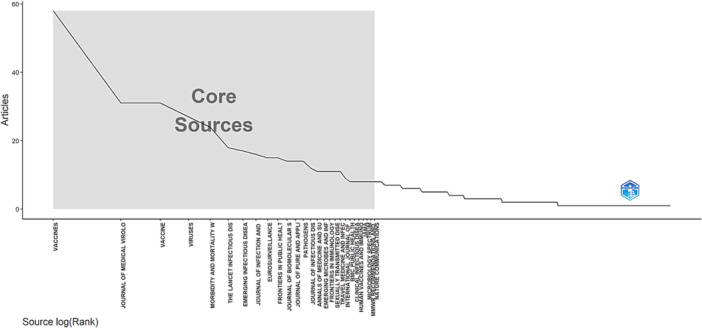
Core sources.

These findings highlight the diversity of sources contributing to the literature on mpox and vaccine research, as well as the importance of these sources in terms of their scholarly impact and relevance to the research community (Figure 1).

**Figure 2:**
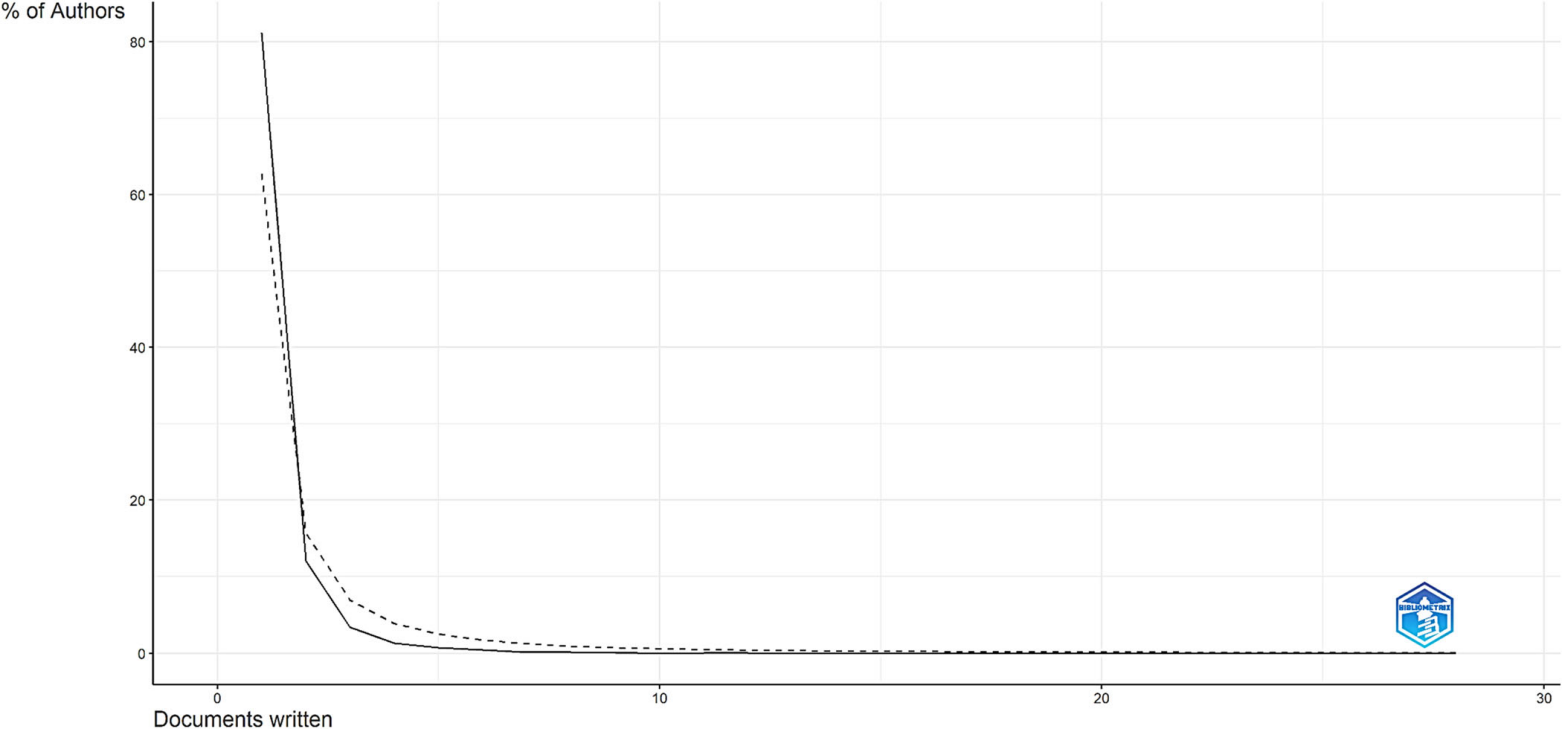
Author productivity.

According to Lotka's law, most authors in the field of mpox and vaccine research have written only one paper. In fact, 6482 authors, representing 81.2% of all authors, have written only one paper. A total of 959 authors (12%) have written two papers, and 272 authors (3.4%) have written three. As the number of papers written by an author increases, the number of authors who have contributed to that number decreases. For example, only 108 authors (1.4%) have written four papers, and only 62 authors (0.8%) have written five. This trend continues until only one author has written 28 papers (Figure 2).

**Figure 3:**
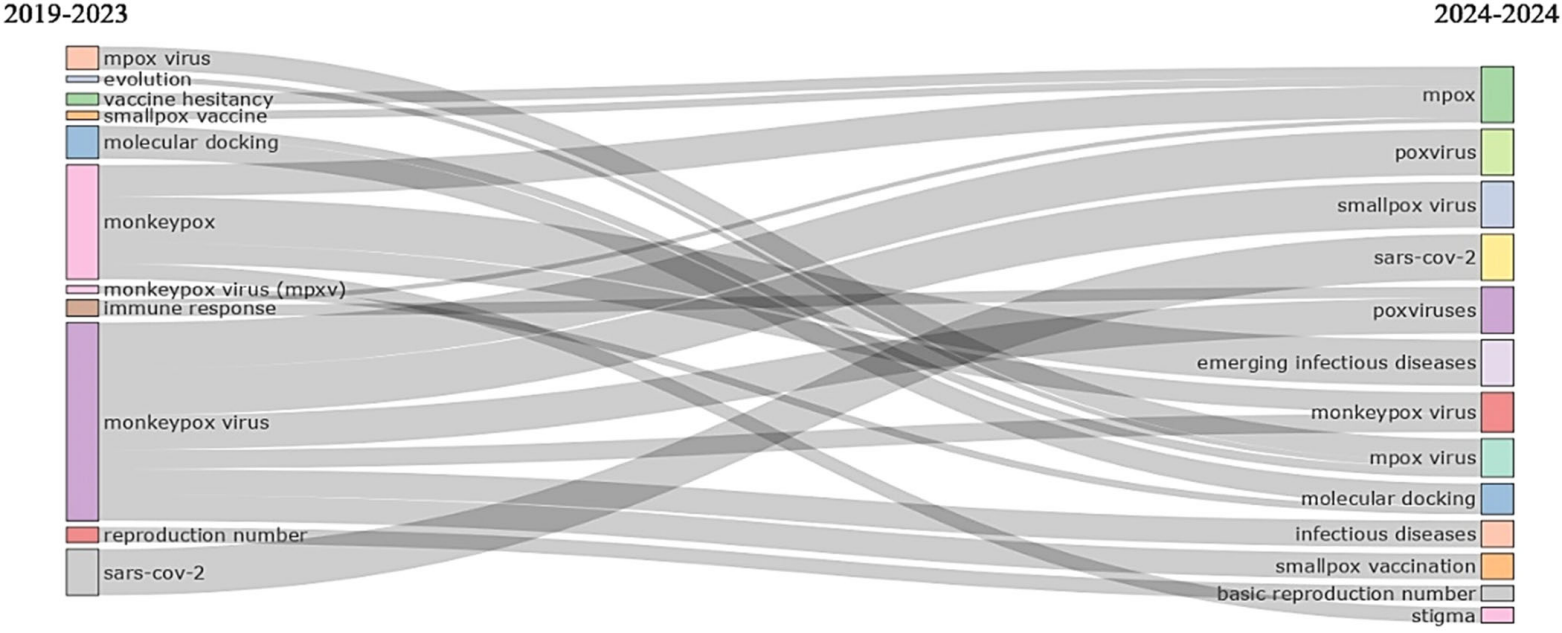
Thematic evolution.

The topic of “evolution” in 2019–2023 became “monkeypox virus” in 2024, with a focus on “monkeypox virus” mentioned 5 times. “Immune response” became ‘monkeypox’ and ‘poxvirus’ in 2024, with an increase in mentions of ‘antibodies’ and ‘neutralizing antibodies’. The theme of “molecular docking” remained constant from 2019 to 2024, but it also became associated with “monkeypox virus” in 2024, highlighting the use of “immunoinformatics” and “reverse vaccinology.” “Monkeypox virus” became ‘infectious diseases’, ‘monkeypox virus’, ‘poxvirus’, ‘pox vaccination’ and ‘smallpox virus’ in 2024, with a focus on ‘monkeypox virus’, ‘orthopoxvirus’, ‘vaccinia virus’, ‘zoonosis’, among others. These results reflect the evolution of research topics in this field and provide valuable insights into emerging areas of interest (Figure 3).

**Figure 4:**
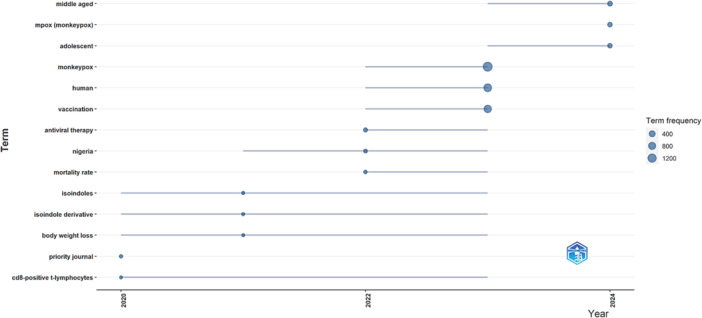
Trend topic.

In 2020, the trending terms were “priority journal” (11 times), “CD8‐positive T lymphocytes” (7 times), “isoindole derivative” (8 times), “isoindoles” (8 times), and “body weight loss” (7 times). Moving forward to 2022 and 2023, the trending terms changed to reflect developments in the field. “Antiviral therapy” (54 times), ‘Nigeria’ (28 times), ‘mortality rate’ (24 times), ‘monkeypox’ (1577 times), ‘human’ (988 times) and ‘vaccination’ (858 times) became key terms. In 2024, trending terms included “median age” (140 times), ‘mpox (monkeypox)’ (121 times), and “adolescent” (120 times) (Figure 4).

**Figure 5:**
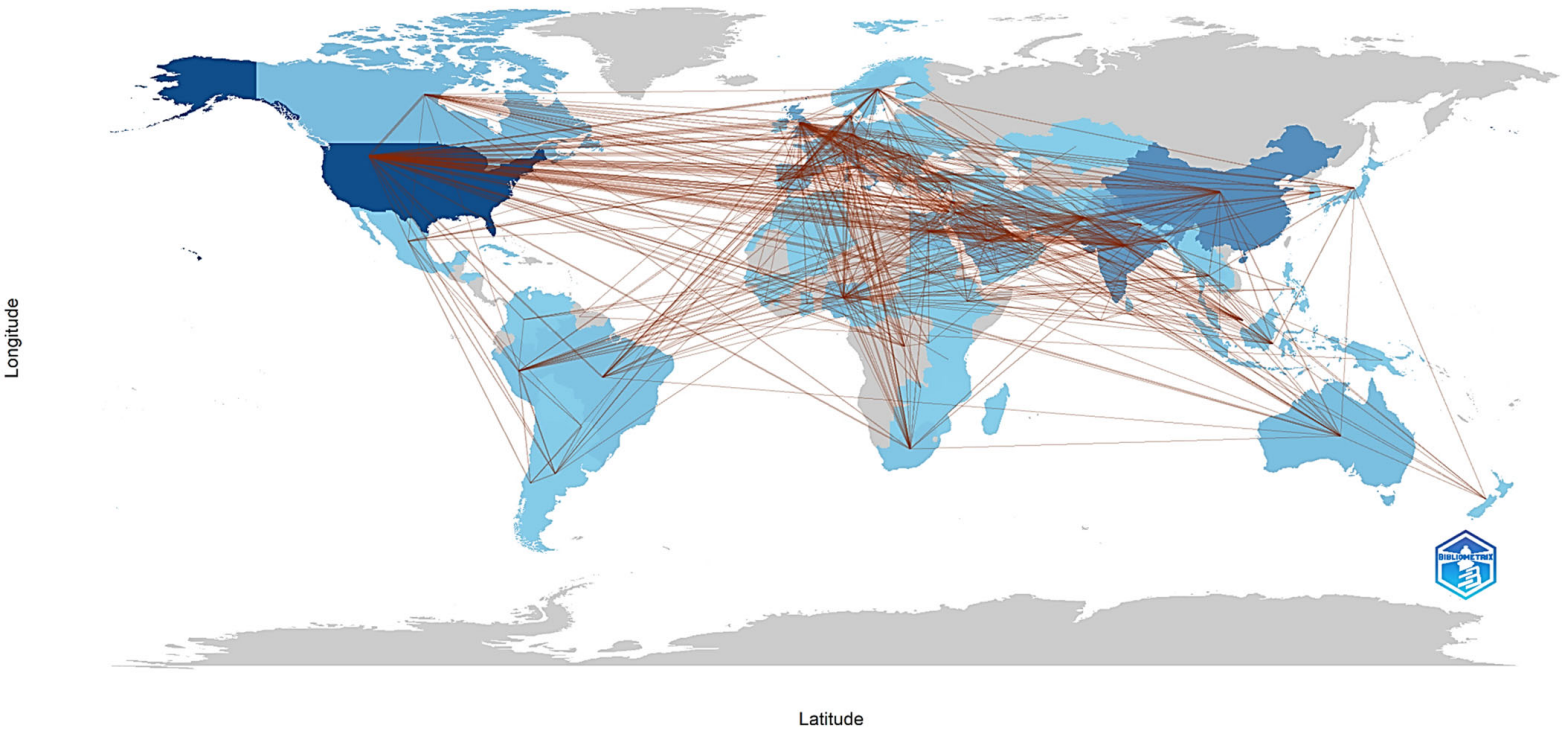
Collaboration map.

According to the map of collaboration between countries, China has the highest number of collaborations with Australia (7 times), Canada (6 times), United Kingdom (6 times), India (5 times), and Saudi Arabia (9 times). Egypt also showed a high frequency of collaboration with Jordan (12 times), Lebanon (9 times) and Nigeria (7 times). In addition, Bangladesh has strong collaborations with Sweden (6 times) and Egypt (7 times). However, Australia has notable collaborations with Sweden (4 times) and Bangladesh (4 times). These data represent the most frequent collaborations between countries (Figure 5).

## 4 Discussion

The results reveal the scientific production of mpox and vaccines. The number of studies published during the search period increased drastically, a situation explained by the outbreak of infections in African countries and the alert issued by the World Health Organization [11]. The need to increase scientific production during an outbreak that quickly became a health alert with the onset of a pandemic generated the development of more research worldwide.

Vaccine research in Mpox is critical to control and prevent the spread of this zoonotic disease. As Mpox resurfaces, the need to develop effective vaccines becomes more urgent. Research in this field is not only focused on creating new vaccines, but also on improving existing vaccines to increase their efficacy and safety. In addition, collaboration between institutions and countries is crucial to share knowledge and resources, thus accelerating the process of vaccine development and delivery. Investment in vaccine research not only has the potential to save lives, but also strengthens the global capacity to respond to future infectious disease outbreaks. Therefore, it is imperative that vaccine research at Mpox receive the attention and resources necessary to advance this critical field of public health. The lack of analysis on the advances, challenges, and collaborations in vaccine research limits a complete understanding of the scientific landscape in this field. To provide a more comprehensive view, it would be beneficial to include an assessment of vaccine studies, highlighting key contributions, emerging trends, and areas requiring further attention. This would not only enrich the study, but also provide a more comprehensive guide for future public health researchers and policy makers [7, 8, 10, 12].

The results indicate that a large proportion of the publications come from institutions that have a great impact on research in general and especially from a country with a constantly growing scientific production [13], the “Center Disease Control”, “Harvard University” and “Johns Hopkins” [14], which indicates leadership in this area of research, possibly due to the academic and work areas they focus on (public health, global health and disease control). However, institutions in other countries, such as King Saud University in Saudi Arabia and Tribhuvan University in Nepal, have also made important contributions. This phenomenon suggests a global interest in mpox research and the distribution of knowledge that can facilitate the implementation of evidence‐based public health policies in various regions [15]. Collaboration between authors from different countries, observed in 31.46% of the articles, underscores an active research network that fosters international cooperation, especially between countries with complementary resources and research needs, as is the case of China and Saudi Arabia or Egypt and Jordan. Countries in Africa and Latin America must also generate collaborative networks despite a global emergency. Collaboration maps show frequent interactions between countries, especially in Asia and the Middle East, pointing to strategic collaboration in regions where zoonoses and emerging infectious diseases represent a considerable burden, as well as having similarities in academic and scientific interests [16]. Partnerships such as those between China and Australia and Canada or between Egypt and Jordan and Nigeria reflect both a focus on mpox research and collaborative efforts to address global public health threats. International collaboration allows decisions to be made on the basis of different populations or to consider similarities between populations while avoiding inequity and fostering inclusiveness [17].

The most influential journals, such as Vaccines and Journal of Medical Virology, not only lead in terms of number of publications but also show significant impact, with high CiteScore and SNIP scores. These journals have been key platforms for the dissemination of recent findings in immunization and monkeypox epidemiology [12], and by the nature of their objectives and scope, they have an audience focused on infectious diseases, epidemiology, and their treatment [18]. Topics highlighted in 2024 include “immune response,” “neutralizing antibodies,” and “reverse vaccinology,” reflecting a focus toward the immunological, biochemical, and host‐interactive understanding of monkeypox [19], which is essential for the development of effective vaccines.

Most authors have contributed a single article, which is common and mediated by Lotka's law [20]. This indicates many researchers in the field, however, there is a small but active core of dedicated experts leading the field, such as Ranjit Sah of Tribhuvan University, who has the largest number of co‐authored publications is Alfonso Rodriguez‐Morales (Fundación Universitaria Autónoma de las Américas, Colombia). Mainly systematic reviews and collaborative narrative reviews are his most cited papers [21, 22]. It is important that collaborative networks are more frequent among authors and countries; Mpox is a global health emergency and responds to public policies mediated by global health [23].

Finally, the search terms reflected significant changes in research trends, emphasizing the key search terms “monkeypox” and “vaccination.” The popularity of terms such as “middle age” and “adolescent” in 2024 suggests an increasing focus on vulnerable populations, possibly due to emerging transmission patterns that require more detailed demographic understanding.

Among the main strengths of the study are the broad coverage and reporting of adequate bibliometric indicators, which allow the analysis of many publications on monkeypox [24, 25, 26, 27], and vaccine research that also have a certain level of quality by the same database to which they are indexed. The observed annual growth rate and the international coauthorship analysis show global interest and collaboration on this topic [28, 29, 30], which is relevant for understanding the spread of knowledge and research priorities. In addition, analysis of keywords and emerging topics allows identification of areas of growing interest, facilitating adaptation of research efforts and health policies.

Among the limitations, the study relies on a specific database, Scopus, which, although comprehensive, does not directly represent research from certain countries, especially developing ones, and that English‐language journals are prioritized, potentially biasing the overall picture. Another limitation is that bibliometric indicators (such as citations and coauthorships) do not adequately reflect the quality or applicability of findings in clinical practice [27] but serve to measure academic impact. Finally, the lack of access to some articles in open or region‐specific repositories could limit the analysis by excluding relevant publications in the field.

Finally, some studies [31, 32] highlight the importance of developing and improving existing vaccines to effectively control Mpox outbreaks. In addition, they underscore the need for international collaboration and a multidisciplinary approach to address the challenges associated with vaccination and disease mitigation. Including these discussions in the study will not only provide a more comprehensive view of vaccine research but will also highlight key areas that require additional attention and resources to advance the fight against Mpox. To strengthen the discussion on mpox vaccine research, it is crucial to address demographic and geographic gaps in the published literature worldwide. Bibliometric analyses of the data not only broaden the understanding of research trends and global spread but also highlight the need for renewed critical interventions in research and health care. Mpox‐related literature does not adequately align with endemic areas, which should be the ideal scenario. These discrepancies between the location of research papers and the endemic epicenters of the disease must be overcome to achieve more effective translation of research findings into public health systems. Addressing these gaps will enable better implementation of vaccination and mitigation strategies, ensuring that scientific advances translate into tangible benefits for the populations most affected by Mpox [28, 29, 30, 31, 32].

## 5 Conclusion

This study allowed us to identify and better understand the key sources, authors, topics, researchers, academics, and policy makers of research in this field. The 91.49% annual growth in papers reflects the growing interest and rapid evolution of research in this field. In addition, the high number of citations per paper indicates the impact and influence of these papers on the scientific community. The diversity of sources and contributing authors highlights the interdisciplinary and collaborative nature of mpox and vaccine research. This is especially relevant in a global context where international collaboration is essential to address public health challenges such as mpox. Finally, by identifying the leading institutions in the field, this study can help guide future collaborations and research efforts. This study is of great utility as it allows us to identify and better understand the key sources, authors, topics, researchers, academics and policy makers in the field of mpox and vaccine research. In addition, the diversity of sources and contributing authors highlights the interdisciplinary and collaborative nature of mpox and vaccine research, which is essential for addressing global public health challenges.

## Author Contributions

**Fran Espinoza‐Carhuancho:** conceptualization, investigation. **Juan Alvitez:** methodology, investigation, conceptualization. **Abigail Temoche:** conceptualization, investigation, methodology. **Victor Roman‐Lazarte:** conceptualization, writing – review and editing, writing – original draft. **Frank Mayta‐Tovalino:** conceptualization, software, formal analysis, writing – review and editing, project administration, resources.

## Conflicts of Interest

The authors declare no conflicts of interest.

## Transparency Statement

The lead author Frank Mayta‐Tovalino affirms that this manuscript is an honest, accurate, and transparent account of the study being reported; that no important aspects of the study have been omitted; and that any discrepancies from the study as planned (and, if relevant, registered) have been explained.

## Data Availability

The data that support the findings of this study are available from the corresponding author upon reasonable request.
